# Comparing endometrial hysteroscopic and histological findings of infertile women with polycystic ovary syndrome and unexplained infertility: A cross-sectional study

**DOI:** 10.18502/ijrm.v18i1.6195

**Published:** 2020-01-27

**Authors:** Sedigheh Amooee, Mojgan Akbarzadeh-Jahromi, Maedeh Motavas, Fatemeh Zarei

**Affiliations:** ^1^Department of Obstetrics and Gynecology, Medical School, Shiraz University of Medical Sciences, Shiraz, Iran.; ^2^Department of Pathology, Medical School, Shiraz University of Medical Sciences, Shiraz, Iran.; ^3^Maternal-fetal Medicine Research Center, Shiraz University of Medical Sciences, Shiraz, Iran.

**Keywords:** Polycystic ovary, Hysteroscopy, Histology, Endometrium, Infertility.

## Abstract

**Background:**

Infertility is a critical condition in women with polycystic ovary syndrome (PCOS), caused not only by anovulation but also by endometrial abnormality.

**Objective:**

This study aimed to evaluate and compare the hysteroscopic and histological findings of endometrial biopsies in infertile women with PCOS and normal endometrial thickness and women with unexplained infertility (UI).

**Materials and Methods:**

This cross-sectional study compared the initial hysteroscopy and endometrial histological findings of 70 infertile women with PCOS and normal endometrial thickness with those of 35 women with UI. The relationship between endometrial histology and clinical parameters such as including luteinizing hormone, follicle-stimulating hormone, thyroid-stimulating hormone, testosterone, prolactin, fasting blood sugar, body mass index (BMI), and infertility duration was analyzed.

**Results:**

The mean age of women with PCOS was significantly lower than that of women with UI (27.5 ± 4.1 vs. 30 ± 4.5 years, respectively) (p < 0.001). The mean BMI was higher in women with PCOS than in women with UI (28.7 ± 4.4 vs. 25.1 ± 3 kg/m${}^{2}$) (p < 0.001). The hysteroscopic findings of all women with PCOS were normal, whereas 91.4% of women with UI had normal hysteroscopic findings, 2.9% had a polyp, and 5.7% had endometrial thickening. The histological findings of women with PCOS revealed proliferative endometrium in 54.3%, disordered proliferative endometrium in 17.1%, secretory endometrium in 8.6%, and endometrial polyp in 17.1%, whereas these percentages in women with UI were 28.6%, 0%, 54.3%, and 20%, respectively.

**Conclusion:**

The hysteroscopic evaluation alone of infertile women might not detect all probable endometrial pathologies in women with PCOS.

## 1. Introduction 

Polycystic ovary syndrome (PCOS) is a highly prevalent endocrine-metabolic disorder in women of reproductive ages, and its estimated global prevalence is 5%-10% (1-3). It is characterized by irregular menstruation/oligomenorrhea, hirsutism, acne, oligo/anovulation, hyperandrogenemia, polycystic ovaries, and infertility (3). According to Rotterdam International Consensus Group, at least two of the following three criteria should be present to diagnose PCOS: oligo/anovulation, elevated levels of circulating androgens or clinical manifestation of androgen excess, and polycystic ovaries on ultrasonography (3, 4). Infertility is a vital concern in women with PCOS, and about 80% of infertile women with anovulatory cycles present with conditions listed in the above diagnostic criteria for PCOS (5). Improvements in ovarian function and ovulation are major challenges for clinicians and researchers in the management of such women, wherein treatment mainly includes medications such as clomiphene citrate, raloxifene, tamoxifen, metformin, aromatase inhibitors, and glucocorticoids as well as surgical management by laparoscopic ovarian drilling (3). Several studies have assessed the etiology of anovulation resulting in infertility in these women; however, studies on the endometrium of women with PCOS are limited (3). Women with improved ovulation through medical therapy reportedly have a higher incidence of implantation failure and spontaneous abortion (3) possibly because of reduced levels of progesterone levels and elevated levels of free insulin levels, insulin growth factor-1, androgens, and luteinizing hormone (LH) during menstrual cycles (3, 6). There is evidence, therefore, suggesting that both anovulation and endometrial abnormality cause infertility in such women (2, 3, 6-8).

Prolonged unopposed estrogen, hyperinsulinemia, and elevated levels of free insulin, growth factor-1, and androgens increase the proliferative activity of the endometrium, resulting in hyperplasia and carcinoma acceleration (3). However, whether women with PCOS without endometrial thickness on ultrasonography require an endometrial biopsy to assess endometrial disorders remains controversial (9).

Therefore, this study aimed to evaluate the endometrium using hysteroscopy and endometrial histology in women with no endometrial thickness on transvaginal ultrasonography and assess its relationship with infertility duration and hormonal patterns in infertile women with PCOS in comparison with women with unexplained infertility (UI).

## 2. Materials and Methods

### Study design

This retrospective cross-sectional study was performed from January 2012 to January 2015. The data obtained from the medical records of patients at the Gynecology Department of Hazrat Zainab Hospital affiliated to Shiraz University of Medical Sciences were retrospectively reviewed and 105 patients were selected.

### Patients

Female patients aged 15-38 yr with a positive history of infertility without any response to ovulation induction therapy and normal semen analyses of their husbands were included in the study. Diagnostic laparoscopy, hysteroscopy, laparoscopic ovarian cautery, and endometrial biopsies were performed in these women. Patients were divided into the following two groups. The PCO group comprised 70 infertile women diagnosed with PCOS, in accordance with the Rotterdam criteria (4), and with normal endometrial thickness on vaginal ultrasonography, normal hysterosalpingographic findings, and no pelvic pathologies such as adhesion and endometriosis on diagnostic laparoscopy. The UI group comprised 35 patients with UI and normal endometrial thickness on vaginal ultrasonography and normal laparoscopic findings. Those who used progesterone supplementation 3-6 months prior to hysteroscopy were excluded.

For both the groups, the exclusion criteria were underlying diseases such as hypertension, diabetes mellitus, cardiovascular diseases, thyroid disorders (patients with abnormal thyroid function tests), hyperprolactinemia, and endometrial lesions, such as leiomyoma and endometrial polyp, on ultrasonography, and positive family history of endometrial cancer.

### Data collection

Demographic data collected from the medical records included age, weight, BMI (weight [kg] divided by height in meters squared [m${}^{2}$]), obstetrics and gynecology history (duration of infertility and variability of menstrual cycles), and medications. Obesity was defined as BMI of >30 kg/m${}^{2}$ and overweight as BMI of >25 kg/m${}^{2}$.

Physical and laboratory examinations (e.g., follicle-stimulating hormone [FSH], LH, thyroid-stimulating hormone [TSH], testosterone, prolactin, fasting blood sugar [FBS], and semen analyses of their husbands) were also performed. Impaired fasting blood sugar was defined as FBS of >100 mg/dL. The FSH and LH levels of the patients were measured on days 3 and 4 of the menstrual cycle. FSH, LH, testosterone, prolactin, and TSH levels were measured using AccuBindⓇ ELISA, Monobind Inc, USA. The normal ranges are as follows: TSH, 0.39-6.16 μIU/mL; FSH, 3.0-12.0 mIU/mL in the follicular phase; LH, 5.0-10.5 mIU/mL in the follicular phase; testosterone, 0.2-0.95ng/mL; and prolactin, 1.2-19.5 ng/mL.

Vaginal ultrasonography (used to report endometrial thickness), hysterosalpingography, laparoscopy, and correlative endometrial biopsy reports were obtained from the patients' medical records.

### Ethical consideration

The study protocol was approved by the Research Ethics Committee of Shiraz University of Medical Sciences (Approval Number: IR.SUMS.REC.1394.S1171). Written informed consent was obtained from the patients.

### Statistical analysis

The SPSS software for Windows version 17 (SPSS Inc. Released 2008, Chicago) was used to analyze statistical data. Descriptive information is presented as mean and standard deviation (SD). In both the groups, hormonal assessment results, including serum levels of TSH, LH, FSH, testosterone, and prolactin, were categorized as high, low, or medium, and Fisher's exact test, chi-square, and Kruskal-Wallis tests were used to analyze qualitative data. The normality of data was analyzed using the Kruskal-Wallis test, and because the clinical parameters did not show a normal distribution, the Mann-Whitney U test was used to compare the quantitative data between the two groups. The contingency and eta coefficients were used to understand the correlations between data. P values of <0.05 were considered statistically significant.

## 3. Results

Comparisons of the PCOS and UI groups revealed that the mean age was significantly lower and BMI was significantly higher in the PCOS group than in the UI group (both p < 0.001) (Table I). The prevalence of obesity and overweight was 37.15% and 47.15% in the PCOS group and 5.7% and 51.45% in the UI group, respectively. The FBS levels were significantly higher in the PCOS group than in UI group (99.20 ± 9.40 vs. 92.90 ± 9.50 mg/mL; p < 0.001). FBS of <100 mg/mL was reported in 49.3% and 74.3% of patients in the PCOS and UI groups, respectively. Patients in the UI group patients had normal TSH, LH, FSH, and testosterone levels, whereas those in the PCOS group had different levels of these hormones, and there were significant differences in LH and testosterone levels between the groups (p < 0.001) (Table II). The results of the Mann-Whitney test showed the LH/FSH ratio was higher in the PCOS group than in the UI group (2.2 ± 1.6 vs. 1.1 ± 0.6, respectively, p < 0.001). Hysteroscopic findings revealed no abnormalities in the PCOS group, where 91.4% of patients in the UI group had normal findings, 5.7% had endometrial thickening, and 2.9% had polyps (p < 0.001). Laparoscopy revealed polycystic ovaries in all the patients in the PCOS group and normal findings in patients of the UI group. There was a significant difference in histological findings between the PCOS and UI groups (p < 0.001) (Table III). The contingency coefficient test, which evaluates the nominal and ordinal variables, revealed no association between hysteroscopic and histological findings (p = 0.28, contingency coefficient = 0.3) and between histological findings and serum FSH, LH, and testosterone levels (p = 0.99, 0.6, and 0.3, respectively). Moreover, the eta coefficient test for the nominal and ordinal variables showed that the histological findings were not associated with age, BMI, and infertility duration (p = 0.2, 0.8, 0.7, and 0.2, respectively).

**Table 1:** The demographic characteristics of patients in the PCO and UI groups


	**PCO group**	**UI group**	**p value**
Age (yr)*	27.50 ± 4.50	30.00 ± 4.10	<0.001
Body mass index (kg/m${}^{2}$)*	28.70 ± 4.40	25.10 ± 3.00	<0.001
Duration of infertility (yr)*	5.20 ± 3.10	3.90 ± 1.90	0.06
Endometrial thickness (mm)*	6.5 ± 1.8	6.6 ± 1.7	0.7
Metformin consumption*	51.5%	5.7%	<0.001
Oligomenorrhea *	14.4%	0	
Oligomenorrhea and hirsutism*	71.7%	0	
Oligomenorrhea and hirsutism and hirsutism *	7.1%	0	
* Data presented as mean ± SD ** Data presented as percentage Chi-square test			

**Table 2:** Comparison of the hormonal levels between the PCO and UI groups


**Hormone **		**UI group**	**PCO group**	**p value**
Follicle-stimulating hormone, pg/ml				
	High	0	1.4	
	Normal	100	97.2	
	Low	0	1.4	0.99
Luteinizing hormone, pg/ml				
	High	0	28.6		
	Normal	100	71.4	
	Low	0	0	<0.001
Thyroid-stimulating hormone, pg/ml				
	High	0	0		
	Normal	100	100	
	Low	0	0	0.99
Testosterone, pg/ml				
	High	0	60.3	
	Normal	100	39.7	
	Low	0	0	<0.001
Prolactin, ng/ml				
	High	0	0	
	Normal	100	100	
	Low	0	0	0.99
Data presented as percentage. Chi-square test				

**Table 3:** Comparison of histological findings between the PCO and UI groups


**Histological findings**	**UI group**	**PCO group**	**p value**
Proliferative endometrium	10 (28.6)	38 (54.3)	
Disordered proliferative endometrium	0 (0)	12 (17.1)	
Secretory endometrium	19 (54.3)	6 (8.6)	
Endometrial polyp	6 (17.1)	14 (20)	<0.001
Data presented as n (%). Chi-square test			

## 4. Discussion 

This study investigated the endometrial abnormality of women with PCOS versus women with UI. The inclusion criteria of both the groups were normal endometrial thickness on vaginal ultrasonography. The results showed that all the women with PCOS had normal hysteroscopic findings but different histological findings. These results indicate that direct visualization by hysteroscopy, known as the gold standard tool for the diagnosis and treatment of infertility and intrauterine cavity abnormalities, may not be sufficient to diagnose endometrial abnormalities in women with PCOS.

Infertility is a major health concern, and PCOS is considered as its common cause (10). Studies investigating the causes of infertility in women with PCOS have mainly focused on anovulation and ovarian dysfunction (3, 11). Endometrial normality is an essential factor in fertility (12); therefore, evaluating the endometrial histology is an important step for identifying endometrial disorders in women with PCOS (2). While hysteroscopy can accurately diagnose endometrial disorders such as endometrial polyps and enable endometrial biopsy (13, 14) with minimal invasion and good tolerance (15), some aspects of hysteroscopy are still controversial (16). Studies have reported the prevalence of minor intrauterine pathologies on hysteroscopy in patients with normal transvaginal ultrasonography to be as high as 20-40% (17, 18). In this study, we investigated women with normal endometrial thickness on transvaginal ultrasonography and performed hysteroscopic endometrial biopsies, which showed different frequencies of endometrial lesions between the PCOS and UI groups: proliferative endometrium (54.3%), disordered proliferative (17.1%), endometrial polyp (20%), and secretory endometrium (8.6%) in the PCOS group, while the most common histological findings included secretory endometrium (54.3%), proliferative endometrium (28.6%), and endometrial polyp (17.1%) in the UI group, with no disordered proliferative endometrium. The high prevalence of endometrial disorders in this study is an important finding considering that previous studies reporting different rates of endometrial hyperplasia in women with PCOS have investigated patients with increased endometrial thickness (19, 20), in whom significant and prolonged increases in estrogen levels can make the patients prone to endometrial polyps, endometrial hyperplasia, and endometrial cancer (19, 21, 22). In our study, exclusion of patients with endometrial thickness revealed no premalignant or malignant lesions in both the PCOS and UI groups despite different endometrial disorders. These results suggest the importance of evaluating endometrial disorders in addition to its association with endometrial thickness, which has been previously demonstrated (2).

Garuti and coworkers matched the hysteroscopic findings with histological findings in a large retrospective study on 1,500 women undergoing diagnostic hysteroscopy and reported endometritis, polyps, endometrial hyperplasia, and endometrial malignancies in 21, 265, 185, and 102 patients, respectively (23). This study suggested the highest sensitivity and specificity of hysteroscopy in the diagnosis of endometrial polyps and the worst sensitivity and specificity in the diagnosis of endometrial hyperplasia (23). Endometrial polyps are benign proliferative lesions, which are incidentally observed on transvaginal ultrasonography, hysterosalpingography, and sonohysterogram (13). Endometrial micropolyps, introduced as small lesions (1-2 mm in length), can only be detected on hysteroscopy (24, 25). Endometrial micropolyps are associated with chronic endometritis (infiltration of plasma cells in the endometrial stroma), endometrial stromal edema, thickening, and periglandular hyperemia (26). A retrospective study reported the estimated prevalence of endometrial micropolyps to be 11% on hysteroscopy using conventional tissue staining and reported its association with endometritis and infertility (24). In the present study, micropolyps were identified in 20% of patients in the PCOS group, although no plasma cells were identified in the micropolyps to diagnose endometritis. These results suggest the importance of detecting micropolyps in women with PCOS, although the identification of plasma cells by conventional tissue staining (e.g., methylgreen-pyronin and hematoxylin-eosin) is not easy even for experienced pathologists because endometrial stromal plasma cells have similar histological features as stromal fibroblasts and mononuclear leukocytes in the endometrium and some morphological characteristics (superficial edema and increased stromal cell density in the secretory phase) may also interfere with the identification of stromal plasma cells (27). Thus, complementary diagnostic techniques such as immunohistochemistry have been suggested for the diagnosis of endometritis (28, 29). The importance of chronic endometritis and micropolyps includes their association with as well as their treatment effects on infertility and endometrial hyperplasia/cancer (30-32).

To the best of our knowledge, no studies have reported the histological findings of women with PCOS with normal hysteroscopic findings. Generally, studies have reported a high correlation between hysteroscopic and histological findings for uterine cavity pathologies (33, 34), while the superiority of histological findings over hysteroscopy and hysteroscopic findings over transvaginal ultrasonographic findings with or without saline infusion for the detection of endometrial lesions have been previously suggested (35). In the present study, there was no association between hysteroscopic and histological findings, suggesting that normal hysteroscopic findings do not indicate normal endometrium and histopathology is required, especially in women with PCOS even without endometrial thickness. Although no premalignant or malignant endometrial lesions were noted in the present study, hysteroscopy without biopsy could not identify endometrial polyps, especially micropolyps detected on histology of endometrial biopsies, possibly because of the absence of endometrial thickening (>12 mm) on transvaginal ultrasonography in both the groups. In assessing endometrial polyps, small nonsessile polyps were one of the limitations in detecting endometrial abnormalities by hysteroscopy.

## 5. Conclusion

The present study revealed that normal hysteroscopic findings were associated with abnormal endometrial biopsies in some infertile women with PCOS without endometrial thickening, suggesting that hysteroscopic evaluation alone of infertile women without histological studies might not be able to detect probable endometrial pathologies such as micropolyps. Therefore, performing hysteroscopic and histological evaluations is recommended in all women with PCOS even in cases with no endometrial thickening or other abnormalities detected on ultrasonography.

##  Conflicts of Interest 

There are no conflicts of interest.
